# Load- and polysaccharide-dependent activation of the Na^+^-type MotPS stator in the *Bacillus subtilis* flagellar motor

**DOI:** 10.1038/srep46081

**Published:** 2017-04-05

**Authors:** Naoya Terahara, Yukina Noguchi, Shuichi Nakamura, Nobunori Kami-ike, Masahiro Ito, Keiichi Namba, Tohru Minamino

**Affiliations:** 1 Graduate School of Frontier Biosciences, Osaka University, 1-3 Yamadaoka, Suita, Osaka 565-0871, Japan; 2 Graduate School of Life Sciences, Toyo University, 1-1-1 Izumino, Itakura-machi, Oura-gun, Gunma 374-0193, Japan; 3 Department of Applied Physics, Tohoku University, 6-6-05 Aoba, Aramaki, Aoba-ku, Sendai, Miyagi 980-8579, Japan; 4 Quantitative Biology Center, RIKEN, 1-3 Yamadaoka, Suita, Osaka 565-0871, Japan

## Abstract

The flagellar motor of *Bacillus subtilis* possesses two distinct H^+^-type MotAB and Na^+^-type MotPS stators. In contrast to the MotAB motor, the MotPS motor functions efficiently at elevated viscosity in the presence of 200 mM NaCl. Here, we analyzed the torque-speed relationship of the *Bacillus* MotAB and MotPS motors over a wide range of external loads. The stall torque of the MotAB and MotPS motors at high load was about 2,200 pN nm and 220 pN nm, respectively. The number of active stators in the MotAB and MotPS motors was estimated to be about ten and one, respectively. However, the number of functional stators in the MotPS motor was increased up to ten with an increase in the concentration of a polysaccharide, Ficoll 400, as well as in the load. The maximum speeds of the MotAB and MotPS motors at low load were about 200 Hz and 50 Hz, respectively, indicating that the rate of the torque-generation cycle of the MotPS motor is 4-fold slower than that of the MotAB motor. Domain exchange experiments showed that the C-terminal periplasmic domain of MotS directly controls the assembly and disassembly dynamics of the MotPS stator in a load- and polysaccharide-dependent manner.

Many motile bacteria can swim in liquid media and move on solid surface by rotating flagella. The bacterial flagellum is composed of the basal body as a rotary motor, the hook as a universal joint, and the filament as a helical propeller. The energy for flagellar rotation is supplied by the electrochemical potential of specific ions across the cytoplasmic membrane. The flagellar motor consists of a rotor and a dozen stators. The rotor is composed of the MS ring made of a transmembrane protein FliF and the C ring consisting of three cytoplasmic proteins, FliG, FliM and FliN. The C ring also acts as a switch of the direction of flagellar motor rotation. In *Escherichia coli* and *Salmonella enterica*, the stator, which is composed of four copies of MotA and two copies of MotB, acts as a H^+^ channel to couple the H^+^ influx through the channel with torque generation. In the Na^+^-driven motor of marine *Vibrio*, PomA and PomB forms a Na^+^ channel complex in a way similar to the MotAB proton channel. Torque is generated by electrostatic interactions of a large cytoplasmic loop in MotA or PomA with FliG^1^,^2^,^3^,^4^,^5^.

More than ten MotAB and PomAB stators can assemble around a rotor through the MotA-FliG and PomA-FliG interactions, respectively^6^,^7^,^8^,^9^,^10^,^11^. The stator is anchored to the peptidoglycan (PG) layer through a well-conserved PG binding motif within the C-terminal periplasmic domain of MotB or PomB^12^,^13^,^14^. High-resolution single molecule imaging techniques have revealed that the MotAB complex rapidly alternates between localized and freely diffusing forms while the motor is rotating^15^. Each stator associates with and dissociates from the motor in response to changes in the environments such as ion motive force across the cytoplasmic membrane and external load^16^,^17^,^18^,^19^,^20^,^21^, suggesting that the flagellar motor acts as a sensor to coordinate the number of active stators in response to the environmental changes. The flagellar motor of *Shewanella oneidensis* MR-1 possesses two distinct types of stators: H^+^-type MotAB and Na^+^-type PomAB. These two stators are incorporated into a motor in response to changes in external Na^+^ concentrations^22^,^23^. A similar observation has been reported in *E. coli* cells that express both the H^+^-type MotAB and Na^+^-type PomA/PotB stators^24^. These suggest that the Na^+^-type stator acts as a Na^+^ sensor to regulate the number of active stators around the rotor in response to external Na^+^ concentrations.

*Bacillus subtilis* too has two distinct H^+^-type MotAB and Na^+^-type MotPS stators although it possesses only a single rotor ring complex (Fig. 1)^25^. The MotAB stator is dominant for wild-type motility under various experimental conditions, because disruption of the *motP* and *motS* genes has no impact on the motility^25^. However, MotPS-dependent motility has been observed at elevated external pH, external Na^+^ concentrations and viscosity when the expression levels of MotP and MotS are considerably increased by a mutation in a stem loop located between the *ccpA* and *motP* genes^26^,^27^. Interestingly, the fully-induced MotPS motor support motility in soft agar at a level comparable to the wild-type and MotAB motors but not in liquid media^25^,^26^. This raises the possibility that the MotPS stator could act as a viscosity or load sensor as well as a Na^+^ sensor. However, it remains unknown how.

Here, we investigated the torque-speed relationships of the MotAB and MotPS motors of *B. subtilis* over a wide range of external loads. We show that the maximum rotation rate of the MotPS motor at low load is about 4-fold slower than that of the MotAB motor but the torque produced by the MotPS stator is nearly the same as that produced by the MotAB stator. The number of active MotPS stators is highly load dependent, making the MotPS motor active only in a viscous environment. We also found that a polysaccharide stabilizes the MotPS stator assembly and increase the motor torque. We will discuss the load- and polysaccharide-dependent activation mechanism of the MotPS motor.

## Results

### Torque-speed relationship of the MotAB and MotPS motors

**Figure 2:**
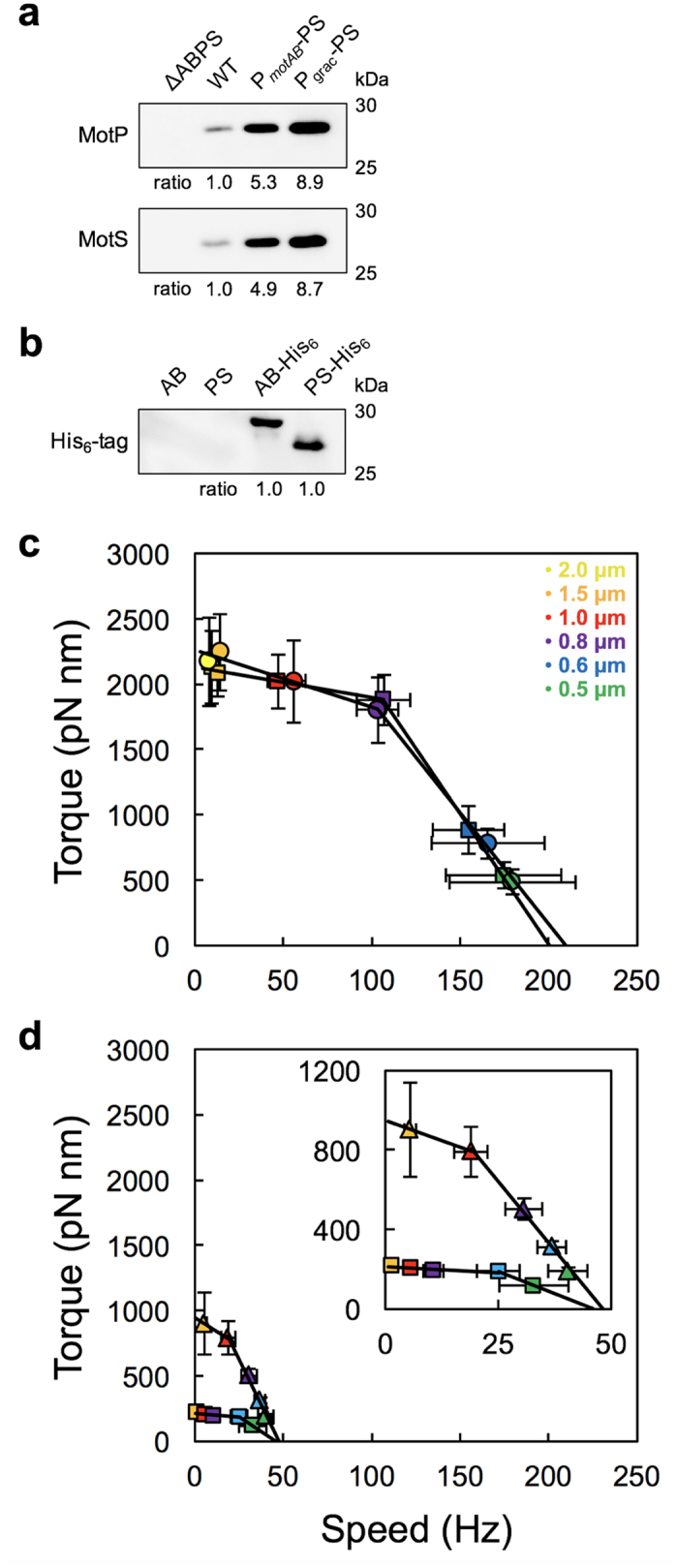
Torque-speed relationship of the *Bacillus* MotAB and MotPS motors. (**a**) Immunoblotting, using polyclonal anti-MotP (upper panel) and anti-MotS (lower panel) antibodies, of the membrane fractions prepared from ∆ABPS, BR151MA (wild type, indicated as WT), P_AB_-PS and P_grac_-PS strains. Each cropped blot was shown by a box. The positions of molecular mass markers were shown on the left. Relative MotP and MotS levels were normalized for wild-type level of each protein. These data were the average of at least three independent experiments. The experimental errors were within 10%. (**b**) Immunoblotting, using monoclonal anti-His-tag antibody, of membrane proteins extracted from the P_AB_-AB, P_AB_-PS, P_AB_-AB-His_6_ and P_AB_-PS-His_6_ strains. The relative level of MotP-His_6_ was normalized for the level of MotB-His_6_. At least three independent experiments were carried out. (**c**) Torque-speed relationship of the MotAB motor under 200 mM K^+^ (circle) or 200 mM Na^+^ (square) condition. Rotation measurements were carried out at 23 °C by tracking the position of 2.0-μm (yellow), 1.5-μm (orange), 1.0-μm (red), 0.8-μm (purple), 0.6-μm (cyan) and 0.5-μm (light green) beads attached to the partially sheared sticky filament. More than twenty individual beads of each size were measured. The curve was fitted by two straight lines with an intersection at the speed of 0.8-μm bead. (**d**) Torque-speed relationship of the MotPS motor. MotP and MotS were expressed from the *P*_*motAB*_ (square) or *P*_*grac*_ promoter (triangle) in the presence of 200 mM NaCl. Rotation measurements were carried out at 23 °C by tracking the position of 1.5-μm, 1.0-μm, 0.8-μm, 0.6-μm and 0.5-μm beads attached to the sticky filament. The curve was fitted by two straight lines with an intersection at the speed of 0.6-μm (square) and 1.0-μm (triangle) bead. The expanded curve is shown in inset.

*B. subtilis* cells expressing only MotPS stator from their own promoter on the chromosomal DNA can hardly swim even in soft agar, because the expression levels of MotP and MotS are too low to drive flagellar rotation^25^,^26^,^27^. To analyze the torque-speed relationship of the Na^+^-driven MotPS motor, we placed the *motP* and *motS* genes under the *P*_*motAB*_ promoter. As a result, the expression levels of MotP and MotS were about 5-fold higher than those in a *B. subtilis* wild-type strain (Fig. 2a). To directly compare the steady cellular level of the MotPS complex expressed from the *P*_*motAB*_ promoter to that of the MotAB complex, we attached a His_6_-tag to a C terminus of MotB or MotS, and then analyzed their expression levels by immunoblotting analysis with monoclonal anti-His_6_-tag antibody (Fig. 2b). The level of MotS-His_6_ (approximately 28 kDa) was essentially the same as that of MotB-His_6_ (approximately 30 kDa).

To clarify any functional differences between the MotAB and MotPS motors, we measured the rotation rates of beads attached to partially sheared sticky filaments of the MotAB and MotPS motors (Fig. 2 and Tables S1 and S2). The torque-speed relationship of the MotAB motor is shown in Fig. 2c. The torque-speed curve of the flagellar motor consists of two regimes: a high-load, low-speed regime and a low-load, high-speed regime^28^,^29^,^30^,^31^,^32^. Consistently, the MotAB motor of *B. subtilis* showed a typical torque-speed curve with a gradual decrease in the high-load regime and a rapid drop in the low-load regime. The zero-speed torque and maximum rotational speed estimated by simple linear extrapolation of the curve in the presence of 200 mM KCl (circle) and NaCl (square) were 2,256 pN nm and 210 Hz, and 2,133 pN nm and 200 Hz, respectively. This indicates that Na^+^ ions do not affect the MotAB motor activity at all. In contrast, the MotPS motor rotated only in the presence of 200 mM NaCl (Fig. 2d, square). The stall torque and maximum rotation speed of the MotPS motor were estimated to be 215 pN nm and 46 Hz, respectively. In the high-load, low-speed regime, the speed of the motor is proportional to the number of active stators in the motor whereas in the low-load, high-speed regime, one stator unit can spin the motor at the maximum speed^33^,^34^. This raises the possibility that the number of active stators is much lower in the MotPS motor than in the MotAB motor.

To clarify this possibility, we next analyzed the multicopy effect of the MotPS stator on the motor performance. To do so, we placed the *motS* and *motP* genes under the control of an isopropyl-β-D-thiogalactopyranoside (IPTG) inducible *P*_*grac*_ promoter. The expression levels of MotP and MotS were about 1.7-fold higher than those transcribed from the *P*_*motAB*_ promoter (Fig. 2a). The zero-speed torque and maximum rotational speed estimated from the torque-speed curve of the MotPS-overproduced motor were 943 pN nm and 48 Hz, respectively (Fig. 2d, triangles), indicating that the overexpression of the MotPS complex increased the zero-speed torque by about 4-fold but did not affect the maximum speed at all. These results suggest that the MotPS stator has a much lower affinity for its binding site of the motor than the MotAB stator. Since high-speed rotation of the flagellar motor at low load is limited by the rate of torque-generation reaction cycle of the motor, we suggest that the maximum activity of the MotPS stator is about 4 times lower than that of the MotAB stator.

### Estimation of the number of stators in the MotAB and MotPS motors

**Figure 3:**
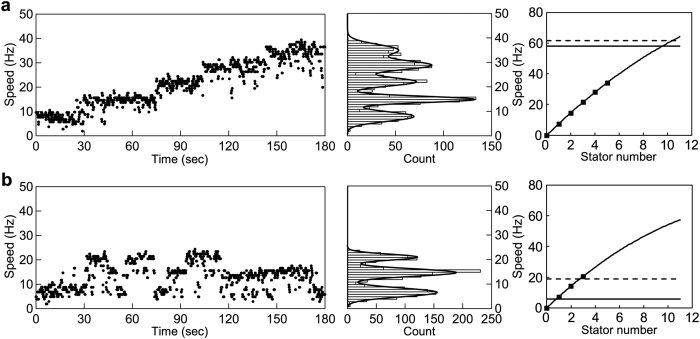
Estimation of the number of active stators in the MotAB and MotPS motors. The (**a**) MotAB or (**b**) MotPS stator was expressed from an IPTG-inducible *P*_*grac*_ promoter by addition of 0.1 mM IPTG. After incubation for 30 min, rotation measurements were carried out at 23 °C by tracking the position of 1.0-μm beads attached to the partially sheared sticky filament in motility buffer containing 1.0 mM IPTG and 200 mM NaCl. The traces and speed histograms are shown on the left and middle. The relationship of the stator number and speed obtained from multiple Gaussian function of speed histograms is shown on the right. A set of data was fitted by second-order polynomial function. The horizontal solid or dash line shows the average speed of each motor when expressed from the *P*_*motAB*_ or *P*_*grac*_ promoter, respectively.

To quantitatively determine the number of active stators in the *Bacillus* MotAB and MotPS motors, we carried out resurrection experiments under high load to measure the stepwise increment in rotation rate upon induction of the MotAB or MotPS stator complex from the IPTG-inducible *P*_*grac*_ promoter. The speed increment in a single MotAB motor with a 1.0-μm bead attached to the partially sheared sticky filament is shown in Fig. 3a. The unit increment was 6–7 Hz as judged by multiple Gaussian fitting of speed histograms (middle panel). Since each increment unit reflects the incorporation of a single stator unit around the rotor, we plotted the stator number versus the motor speed and fitted a set of data by a second-order polynomial function. Because the average speed of the MotAB motor with a 1.0-μm bead attached was about 60 Hz (Fig. 2c), the number of active stators in the MotAB motor was estimated to be about ten. Similar stepwise increments were observed in the MotPS motor as well (Fig. 3b). The increment unit (6–7 Hz) was essentially the same as that of the MotAB motor, indicating that a single MotPS stator unit produces nearly the same torque as a single MotAB stator unit. This suggests that the energy coupling efficiency of the MotPS stator is essentially the same as that of the MotAB stator. In contrast to the MotAB motor, however, accelerations and decelerations of motor rotation are more frequently observed in the MotPS motor (Fig. 3b, left panel), indicating that the dissociation rate constant of the MotPS stator from its binding site on the motor is much higher than that of the MotAB stator. Since the average speeds of the MotPS motors with 1.0-μm beads attached were about 6.2 Hz and 18 Hz when expressed from the *P*_*motAB*_ (horizontal solid line) and *P*_*grac*_ promoters (horizontal dash line), respectively, the number of active stators in the MotPS motor were estimated to be about one to three.

### Motor performance of the MotPS motor in the presence of Ficoll 400

**Figure 4:**
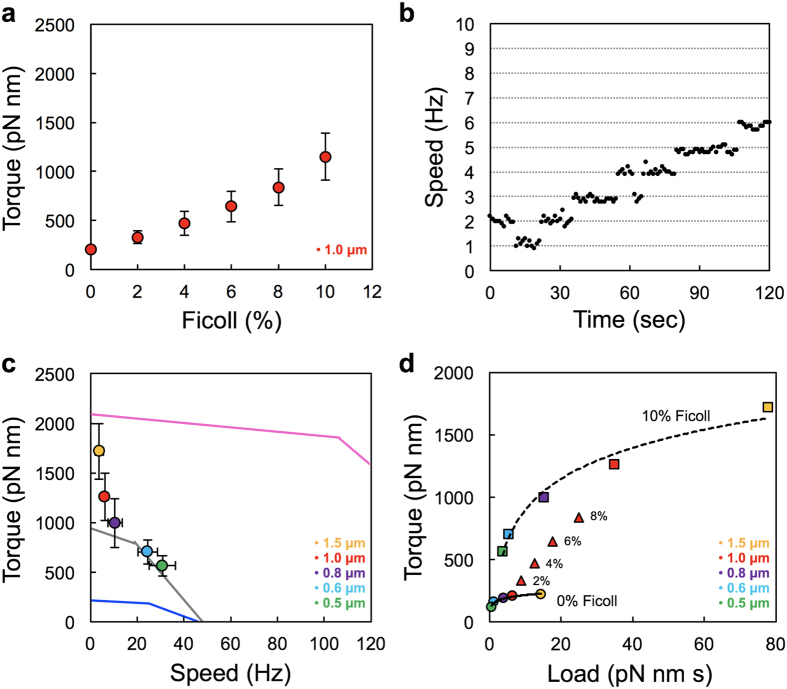
Rotational measurements of the MotPS motor under high viscous conditions. (**a**) Torque of the MotPS motor measured using a 1.0-μm bead in media containing 0%, 2%, 4%, 6%, 8% and 10% Ficoll 400 (w/v). (**b**) Speed increment in a single MotPS motor with a 1.0-μm bead attached in the presence of 10% Ficoll. The expression of the MotPS stator was induced from an IPTG-inducible *P*_*grac*_ promoter by addition of 0.1 mM IPTG, followed by incubation at 37 °C for 30 min and finally rotation measurements at 23 °C by tracking the position of 1.0-μm beads attached to the partially sheared sticky filament in motility buffer containing 1.0 mM IPTG, 200 mM NaCl and 10% Ficoll. (**c**) Torque-speed relationship of the MotPS motor in the presence of 10% Ficoll 400 (w/v) Rotation measurements were carried out at 23 °C by tracking the position of 1.5-μm (orange), 1.0-μm (red), 0.8-μm (purple), 0.6-μm (cyan) and 0.5-μm (light green) beads attached to the sticky filament. At least twenty individual beads were measured. Lines indicate the torque-speed curves of the MotAB (magenta) and MotPS motors (blue and grey) shown in Fig. 2. Blue and grey lines indicate the torque-speed curves of the MotPS motors, which were expressed from the *P*_*motAB*_ and *P*_*grac*_ promoter, respectively. (**d**) Effect of Ficoll 400 on torque generation by the MotPS motor. Torque versus frictional drag coefficient are plotted and fitted by second-order polynomial function. Squares and circles indicate two sets of data of the MotPS motor labelled with 1.5-μm (orange), 1.0-μm (red), 0.8-μm (purple), 0.6-μm (cyan) or 0.5-μm (light green) bead in the presence and absence of 10% Ficoll 400. Triangles show a set of data of the MotPS motor labelled with a 1.0-μm bead in media containing 2%, 4%, 6% and 8% Ficoll 400 (w/v).

Considerable MotPS-dependent motility of *B. subtilis* has been observed in soft agar but not in liquid media^25^,^26^, raising the possibility that an increase in viscosity enhances the MotPS stator activity. To address this, we measured the rotation speed of a single 1.0 μm bead attached to a partially sheared sticky filament of the MotPS motor in motility buffers containing Ficoll 400 over a range of concentrations from 0% to 10% (w/v) in the presence of 200 mM NaCl (Fig. 4a and Table S3). As the concentration of Ficoll 400 was increased step-by-step by 2% at each step, the motor speed did not change at all, indicating that torque was increased (Table S3). The torque calculated from the rotation rate and drag force was 209 ± 35 pN nm at 0% Ficoll and was increased to 1150 ± 238 pN nm at 10% Ficoll (Fig. 4a), raising the possibility that the number of active stators in the motor was increased with an increase in the Ficoll concentration. To clarify this, we carried out resurrection experiments in the presence of 10% Ficoll. The unit increment in the rotation speed of a single MotPS motor with a 1.0-μm bead attached was about 1 Hz (Fig. 4b), and the torque produced by a single MotPS stator unit was estimated from this to be about 210 pN nm, which is almost the same torque produced by a single MotPS stator unit in the absence of Ficoll. These results indicate that the number of active stators is 5 to 6 at 10% Ficoll and that Ficoll 400 facilitates the assembly of the MotPS stators around the rotor in a concentration-dependent manner.

We next analyzed the torque-speed relationship of the MotPS motor in the presence of 10% Ficoll 400 (Fig. 4c and Table S4). The MotPS motor showed an unusual torque-speed curve with a rapid drop in the high-load regime and a slow decrease in the low-load regime. The torque at extremely high load (1.5-μm bead) was about 1,717 ± 279 pN nm at a speed of around 3.8 Hz, and then the torque decreased rapidly to 1,260 ± 238 pN nm at around 6.2 Hz (1.0-μm bead) and further down to 995 ± 248 pN nm at around 10.5 Hz (0.8-μm bead). Then, the torque showed a slow decrease from 704 ± 118 (0.6-μm bead) to 563 ± 102 pN nm (0.5-μm bead) over a speed range from 24.4 to 30.8 Hz. The stall torque produced by the MotPS motor in the presence of 10% Ficoll was estimated by extrapolation to be about 2,200 pN nm, which is almost the same as that of the MotAB motor. These results suggest that a high load stabilizes the interaction of each MotPS stator unit with its binding site on the motor and that only a small decrease in the load induces rapid dissociation of several MotPS stators from the motor.

Interestingly, the torque produced by the MotPS motor was much larger in the presence of Ficoll than in its absence even under the same load and showed a dependence on the Ficoll concentration (Fig. 4d). This suggests that not only the external load but also Ficoll 400 itself as a chemical facilitates the MotPS motor performance by stabilizing the MotPS stator assembly around the rotor. A much more stable increment in the speed of the MotPS motor in the resurrection experiment in the present of 10% Ficoll (Fig. 4b) compared to the large speed fluctuation in the absence of Ficoll (Fig. 3b) also indicate such a stabilizing effect on the active MotPS stator by this polysaccharide.

### Effect of domain exchange between MotAB and MotPS on the motor performance

**Figure 5:**
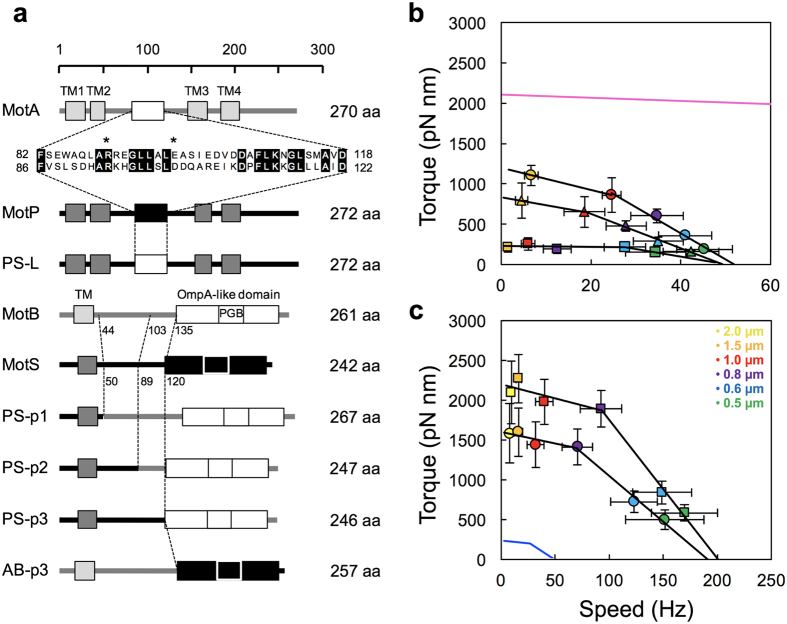
Effect of domain exchange between the MotAB and MotPS stators on motor performance of the *Bacillus* motor. (**a**) MotA and MotP have four transmembrane (TM) helices and a large cytoplasmic loop between TM2 and TM3. The PS-L motor was constructed by exchanging the cytoplasmic loop of MotP with that of MotA. Conserved residues and critical charged residues are shown by black boxes and asterisks, respectively^45^. MotB and MotS have a single TM helix and a C-terminal large periplasmic domain containing a PG binding motif (OmpA-like domain), which is critical for stator assembly around the rotor^46^. The PS-p1, PS-p2 and PS-p3 motors were constructed by the exchange of the indicated region of MotS with the corresponding region of MotB. The AB-p3 motor was constructed by exchanging the OmpA-like domain of MotB with that of MotS. (**b**) Torque-speed relationship of the PS-L (square), PS-p2 (triangle) and PS-p3 (circle) motors in the presence of 200 mM NaCl. Rotation measurements were done by tracking the position of 1.5-μm, 1.0-μm, 0.8-μm, 0.6-μm and 0.5-μm beads. The torque-speed curve of the MotAB motor is shown by magenta line. (**c**) Torque-speed relationship of the AB-p3 motor in the presence of 200 mM NaCl (square) or KCl (circle) condition. Rotation measurements were carried out by tracking the position of 2.0-μm, 1.5-μm, 1.0-μm, 0.8-μm, 0.6-μm and 0.5-μm beads. The torque-speed curve of the MotPS motor is shown by blue line.

It has been shown that the C-terminal periplasmic domain of MotB (MotB_C_) is critical for load-dependent changes in the number of active stators in the flagellar motor^19^. Electrostatic interactions of the cytoplasmic loop of MotA with FliG are important not only for torque generation but also for efficient stator assembly around a rotor^7^,^8^. To clarify which domain of the MotPS stator contribute to a viscosity-dependent change in the stator number in the motor, we carried out domain exchange experiments. To do so, we constructed five chimeras: PS-L, PS-p1, PS-p2, PS-p3 and AB-p3 (Fig. 5a). The PS-L, PS-p2, PS-p3 and AB-p3 chimera stators were functional whereas the PS-p1 stator was not (Fig. 5b,c and Table S5). The stall torque of the PS-p2 (Fig. 5b, triangle) and PS-p3 motors (circle), in which residues 89–242 and 120–242 of MotS were replaced by residues 103–261 and 135–261 of MotB, respectively, were about 4-fold and 6-fold higher than that of the MotPS motor, respectively. However, the maximum speeds of the PS-p2 and PS-p3 chimera motors at low load were around 50 Hz, almost the same as that of the MotPS motor. In contrast, the stall torque and maximum rotational speed of the PS-L motor, in which residues 86–122 of MotP were replaced by residues 82–118 of MotA, were almost the same as those of the MotPS motor (Fig. 5b, square). These results suggest that the C-terminal periplasmic domain of MotS (MotS_C_) is responsible for regulating the number of active stators in the MotPS motor in response to changes in the external load and that the cytoplasmic loop of MotP does not contribute to the binding affinity of the MotPS stator for the motor.

The AB-p3 chimera motor, in which residues 135–261 of MotB_C_ was replaced by residues 120–242 of MotS_C_ (Fig. 5a), showed the same torque-speed curve in the presence of 200 mM NaCl as the MotAB motor (Fig. 5c, square and Table S6). Since residues 135–261 of MotB_C_ and residues 120–242 of MotS_C_ form a OmpA-like domain, which binds to the PG layer (Fig. 5a), this suggests that the binding affinity of MotS_C_ for the PG layer is the same as that of MotB_C_. Therefore, we propose that residues 50–120 of MotS_C_ is a load-dependent structural switch to control the binding affinity of MotS_C_ for the PG layer in response to changes in viscosity. Interestingly, the stall torque produced by the AB-p3 motor was lower in the presence of 200 mM KCl (Fig. 5c, circle) than in the presence of 200 mM NaCl (square) whereas the maximum rotational speed of the AB-p3 motor was the same under these two conditions. This result suggests that Na^+^ ions stabilize the association of MotS_C_ with the PG layer.

## Discussion

The bacterial flagellum is not only supramolecular motility machinery for bacterial locomotion through liquid media and across solid surfaces, but also a mechanosensor that detects changes in the environment, thereby inducing cell differentiation and hyper-flagellation^5^. The *E. coli* and *Salmonella* flagellar motor regulates the number of active stators in the motor in response to changes in the external load^16^,^17^,^19^. Wild-type *B. subtilis* has two distinct types of stators: H^+^-type MotAB and Na^+^-type MotPS^25^. The MotAB stator is a major contributor for torque generation by the *B. subtilis* motor because disruption of the MotPS stator does not affect motility significantly under various experimental conditions. The MotPS stator can support Na^+^-dependent motility when over-expressed^25^,^27^. Interestingly, the Na^+^-dependent motility of the MotPS-overexpressing strain in soft agar is almost the same as that of the *B. subtilis* wild-type and MotAB-expressing strains. However, free-swimming speed of the MotPS-overexpressing cells is about 5-fold slower than those of the wild-type and MotAB motile cells^26^.

To clarify whether the MotPS motor activity depends on high viscosity, we measured the rotational speeds of the MotAB and MotPS motors over a wide range of external loads by bead assays with or without Ficoll 400 and showed that the number of active stators in the MotPS motor is increased from one to ten with an increase in the concentration of Ficoll 400 (Fig. 4). The MotAB motor contained about ten active stator units around the rotor at high load (Figs 2c and 3a). In contrast, when the MotPS stator was expressed from the *motAB* promoter, the MotPS motor contained only one active stator unit around the rotor (Fig. 2d) although the expression level of the MotPS complex was essentially the same as that of the MotAB complex (Fig. 2b). But when the expression level of the MotPS complex was increased by 2-fold, the stall torque was increased from 220 to 943 pN nm due to an increase in the number of active stators in the motor from one to three (Figs 2 and 3). Accelerations and decelerations of motor rotation of the MotPS motor were frequently observed in the resurrection experiments, indicating that the MotPS stator dissociates from and associate with the rotor more frequently than the MotAB stator. Therefore, we suggest that the binding affinity of the MotPS stator for the motor is about 10-fold lower than that of the MotAB stator in the absence of Ficoll. A stepwise increment in the Ficoll concentration linearly increased the torque produced by the MotPS motor due to an increase in the number of active MotPS stators in the motor (Fig. 4a and b). The maximum stall torque of the MotPS motor was estimated to be about 2,200 pN nm, which was the same as that of the fully installed MotAB motor (Fig. 4c). This indicates that up to about ten MotPS stators are incorporated into the motor in a Ficoll concentration-dependent manner. However, the torque drastically dropped off by a slight decrease in the external load and then showed a slow exponential decay over a wide range of load by its further reduction (Fig. 4c). Since the number of active stators changes in a load-dependent manner^17^,^18^,^19^, we suggest that this unusual torque-speed relationship of the MotPS motor is a consequence of its quite high mechanosensitivity. However, since Ficoll 400 showed another effect on the MotPS motor activity, directly activating the motor in a concentration-dependent manner even at the same load (Fig. 4d), we suggest that the MotPS stator is also a polysaccharide sensor that detects changes in the polysaccharide concentration in the environment. It has been reported that the MotPS stator contributes to biofilm formation^25^. Biofilm is composed of the matrix of extracellular polysaccharide. Since Ficoll 400 is a neutral, highly branched, hydrophilic polysaccharide, we propose that the MotPS stator may considerably contribute to the motility of wild-type *B. subtilis* cells in biofilm formation. We are currently testing this hypothesis.

In-frame deletion of residues 72–100 in MotB_C_ causes a rapid decrease in the number of stators at a motor load much higher than that at which the wild-type stator begins to dissociate from the motor, and it has been proposed that MotB_C_ may regulate the assembly and disassembly dynamics of the stators in response to load changes^19^. The replacement of the OmpA domain of MotS_C_ by that of MotB_C_ increased the stall torque by about 6-fold (Fig. 5a and b). Because MotB_C_ and MotS_C_ are responsible for stable anchoring of the stator to its binding-site in the motor, we suggest that the binding affinity of MotS_C_ for its binding site in the motor is significantly lower than that of MotB_C_ and that Ficoll 400 stabilizes the association of MotS_C_ with its binding site in the motor as well as with the PG layer. A linker region between TM-1 and the OmpA-like domain of MotS appear to contribute to its binding affinity of MotS_C_ for the PG layer in a load- and polysaccharide-dependent manner (Fig. 5b), suggesting that the linker region of MotS acts as a structural switch to regulate the assembly and disassembly dynamics of the MotPS stator in response to load changes as proposed previously^19^. In the presence of 200 mM NaCl, the AB-p3 motor, in which the OmpA-like domain in MotB_C_ was replaced by that in MotS_C_, showed the same torque-speed curve as the MotAB motor (Fig. 5c). Interestingly, the stall torque produced by the AB-p3 motor decreased significantly in the absence of NaCl whereas the zero-torque speed was the same as that in the presence of NaCl. Since the OmpA-like domain contains the PG binding motif, we suggest that MotS_C_ has the Na^+^ binding site to stabilize its association with the PG layer.

The near zero-load speed of the MotAB motor was ca. 200 Hz (Fig. 2c), whereas that of the MotPS motor was about 50 Hz (Fig. 2d). The flagellar motor containing a single stator unit shows 26 steps per revolution, and each step consists of at least two distinct processes: a torque generation step involving stator-rotor interactions and a step for recovery of the original stator-rotor geometry^35^,^36^. Since the maximum rotation speed of the motor is limited by the rate of torque generation cycle of the motor, the rate of conformational changes of the MotPS stator coupled with the Na^+^ flow is about 4-fold slower than that of the MotAB stator coupled with the H^+^ flow. It has been shown that the Na^+^-type PomA/PotB motor can rotate at about 200 Hz under low load in the presence of 85 mM NaCl but the low-load speed falls down to about 50 Hz when the external Na^+^ concentration is decreased from 85 to 1 mM. This indicates that the Na^+^ concentration gradient across the membrane limits the motor speed of the PomA/PotB motor^37^. A single MotPS stator unit generated nearly the same torque as a single MotAB stator unit when operating under high load (Fig. 3), indicating that the energy coupling efficiency of the MotPS stator is the same as that of the MotAB stator. Since the maximum speed of the MotPS motor is about 50 Hz even in the presence of 200 mM NaCl, either Na^+^ transit through the channel of the MotPS stator, Na^+^ release from the channel to the cytoplasm or both may limit the rate of torque generation cycle of the MotPS motor.

Many molecular motors sense force to coordinate their biological activity. Myosin, which is an ATP-driven liner motor, alters its ATPase activity and mechanical properties in response to changes in the external load^38^,^39^. It has been shown that the flagellar motor regulates not only the number of active stators in the motor but also the proton channel activity of each stator in response to load changes^16^,^17^,^18^,^19^,^20^. Interestingly, changes in the bacterial flagellar motor activity lead to cell differentiation in response to changes in environments^40^. Since the MotPS motor is a load and polysaccharide sensor as well as a Na^+^ sensor, our present results provide a new insight into the mechanism of how *B. subtilis* cells sense and adopt to changes in environments. Furthermore, because MotSc has a structural switch to regulate the assembly and disassembly dynamics of the MotPS stator in a load- and polysaccharide dependent manner, it will be a useful model for molecular design of a load-sensing actuator in the future.

## Methods

### Bacterial strains and growth conditions

Bacterial strains used in this study are listed in Table S7. *E. coli, B. subtilis* and its derivatives were routinely cultured at 37 °C in Luria Broth (LB) containing 1% Bacto tryptone, 0.5% Yeast extract, 0.5% NaCl. Ampicillin, chloramphenicol, erythromycin, neomycin and spectinomycin were added at 50 μg/ml, 5 μg/ml, 0.3 μg/ml, 7.5 μg/ml and 150 μg/ml, respectively if necessary. Motility buffer contained 10 mM potassium phosphate, pH 7.0, 0.1 mM EDTA, 10 mM L-lactic acid.

### Strain and plasmid constructions

All plasmids used and constructed in this study are listed in Table S8. All primers used for constructions of strains and plasmids are shown in Table S9. Recombinant DNA manipulations were performed using standard protocols.

To attach a His_6_-tag to the C-terminus of MotB or MotS, oligonucleotides that contain sequence encoding a His_6_-tag at the 3′ end of *motB* or *motS* were designed (Table S9). The *motAB-his*_*6*_ and *motPS-his*_*6*_ genes was amplified by PCR using pDR-AB and pDR-PS as templates, respectively. The PCR products of the *motAB-his*_*6*_ and *motPS-his*_*6*_ genes were digested with *Xma*I and *Sph*I and cloned into the *Xma*I and *Sph*I sites of an integration plasmid pDR67, yielding pDR-AB-His_6_ and pDR-PS-His_6_, respectively. To put *motAB* and *motPS* under an IPTG-inducible *P*_*grac*_ promoter, the *motAB* and *motPS* genes were amplified by PCR using the *B. subtilis* chromosomal DNA as a template with primers AB-*Bam*HI-F and AB-*Xma*I-R and primers PS-*Bam*HI-F and PS-*Xma*I-R, respectively. The PCR products containing the *motAB* and *motPS* genes were digested with *Bam*HI and *Xma*I and cloned into the *Bam*HI and *Xma*I sites of an expression plasmid pHT01, yielding pHT-AB and pHT-PS, respectively. To subclone these two stator genes into the pDR67 integration vector, the P_*grac*_-*motAB* and P_*grac*_-*motPS* genes were amplified by PCR using pHT-AB and pHT-PS as templates with primers P_*grac*_-*Xma*I-F and AB-*Sph*I-R and P_*grac*_-*Xma*I-F and PS-*Sph*I-R, respectively. The PCR products were digested with *Xma*I and *Sph*I and cloned into the *Xma*I and *Sph*I sites of pDR67, yielding pDR-P_*grac*_-AB and pDR-P_*grac*_-PS.

To replace the *hag* gene by the *spec*^*r*^ gene, approximately 500 bp DNA fragments corresponding to each side of the *hag* gene were amplified by PCR using *B. subtilis* chromosomal DNA as a template with primers Hag-del-F1 and Hag-del-R2 for the upstream region and primers Hag-del-F3 and Hag-del-R1 for the downstream region, respectively. The *spec*^*r*^ gene was also amplified by PCR using a plasmid pDG1730 as a template with primers Hag-del-F2 and Hag-del-R3. The amplified fragment was directly used to delete the *hag* gene on the chromosome. Recombinants were selected as a spectinomycin-resistant colony, and the expected deletions were confirmed by DNA sequencing.

To allow polystyrene beads to be efficiently and rapidly attached to the flagellar filaments of *B. subtilis*, we introduced the D2 domain (142–203 and 293–395) of *Salmonella* FliC(∆204–292) that forms sticky filaments^41^,^42^. The *hag* gene was amplified by PCR using the *B. subtilis* chromosomal DNA as a template with primers Hag-*Bam*HI-F and Hag-sticky-R1 for the region coding the *hag* gene promoter and the N-terminal segment (1–141) and primers Hag-sticky-F2 and Hag-*Sph*I-R for the region coding the C-terminal (215–304), respectively. The D2 domain of *Salmonella* FliC(∆204–292) was also amplified by PCR using the chromosomal DNA of the *Salmonella* SJW46 strain^41^ as a template with primers Hag-sticky-F1 and Hag-sticky-R2. These three PCR products were joined by overlapping PCR. The PCR product was digested with *Bam*HI and *Sph*I and cloned into the *Bam*HI and *Sph*I sites of the pDR67 vector, yielding pDR-hagsticky.

To co-express the MotAB and MotPS stators together with the sticky flagellar filament, each stator genes were extracted from each plasmid by digesting with and subcloned into the *Xma*I and *Sph*I sites of the pDR-hagsticky plasmid. To construct the domain-exchanged stator genes (AB-p3, PS-L, PS-p1, PS-p2 and PS-p3), each domain of the MotAB and MotPS stators was amplified by PCR using pDR-AB and pDR-PS as templates with primers AB-*Xma*I-F and PS-*Sph*I-R for AB-p3 and PS-L and primers AB-*Xma*I-F and AB-*Sph*I-R for PS-p1, PS-p2 and PS-p3, respectively. These PCR products were joined by PCR reaction and then inserted into the pDR-hagsticky plasmid. When all pDR67-based plasmids were introduced to *B. subtilis*, recombinant strains were selected as a chloramphenicol-resistant and amylase-negative phenotype.

### Immunoblotting analysis

To investigate the expression level of each stator protein in the cytoplasmic membrane, *B. subtilis* cells were grown at 37 °C for 5 h with shaking, harvested and washed with 50 mM Tris-HCl, pH 8.0. The cells were suspended in 50 mM Tris-HCl, pH 8.0 with DNase I. Membrane vesicles were prepared using a FRENCH pressure cell (FA-032, Central Scientific Commerce). Undisrupted cells were removed by low-speed centrifugation. Then, membrane vesicles were collected by ultracentrifugation and suspended in 50 mM Tris-HCl, pH 8.0. The protein concentration of the membrane fraction was measured by the Lowry method. 10 μg of membrane protein from each sample was used for sodium dodecyl sulfate-polyacrylamide gel electrophoresis (SDS-PAGE) and immunoblotting with polyclonal anti-MotP, anti-MotS and anti-His_6_-tag antibody was carried out as described previously^27^,^43^.

### Bead assay

*Bacillus* strains producing sticky filaments were grown at 37 °C with shaking until the cell density had reached an optical density at 600 nm of 1.0. The sticky flagellar filaments were sheared by passing through a 25-gauge needle, and then the cells were attached to the surface of a cover slip. Polystyrene beads with diameters of 2.0 μm, 1.5 μm, 1.0 μm, 0.8 μm, 0.6 μm or 0.5 μm (Invitrogen) were attached to the filament. Bead assays were carried out at 23 °C in motility buffer containing 200 mM KCl or 200 mM NaCl with or without Ficoll-PM400. Phase contrast images of each bead were captured by a high-speed camera (ICL-B0620M-KC; Imperx) at the frame rate of 1,000 frames per second as described^19^. The rotation speed analysis and torque calculation were carried out as described^19^,^42^.

In resurrection experiments, the cultured cells were suspended in fresh medium containing 0.1 mM IPTG and incubated for 30 min at 37 °C with shaking. After shearing the filament, bead assay was carried out in motility buffer containing 1.0 mM IPTG and 1.0 μm beads. The rotation speed was measured by projecting the phase contrast image of each bead onto a quadrant photodiode through a 60× oil immersion objective lens^40^. All data were recorded at 1.0 ms intervals by a 16-bit A-D board (Microscience) and the LaBDAQ software (Matsuyama Advance), and analyzed as described previously^36^.

## Additional Information

**How to cite this article**: Terahara, N. *et al*. Load- and polysaccharide-dependent activation of the Na^+^-type MotPS stator in the *Bacillus subtilis* flagellar motor. *Sci. Rep.* **7**, 46081; doi: 10.1038/srep46081 (2017).

**Publisher's note:** Springer Nature remains neutral with regard to jurisdictional claims in published maps and institutional affiliations.

## Supplementary Material

Supplementary Information

## Figures and Tables

**Figure 1:**
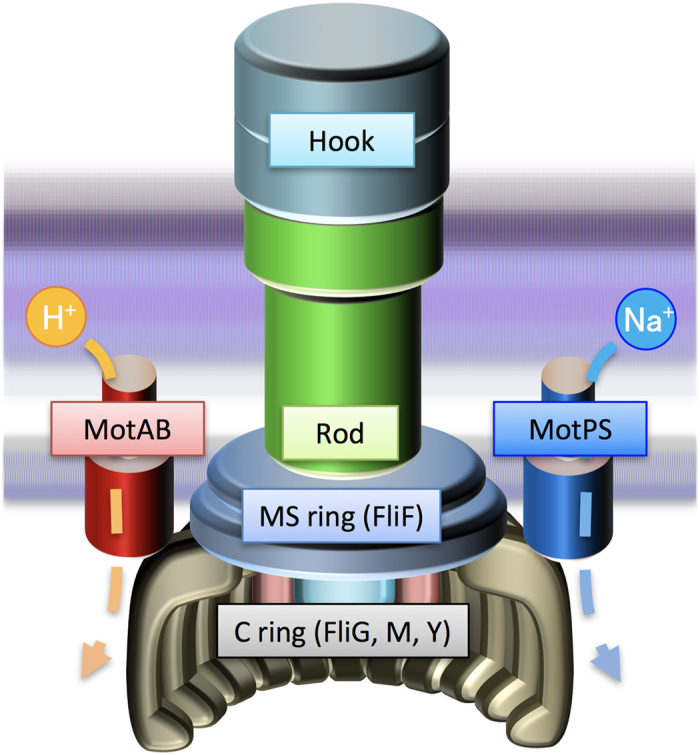
Schematic diagram of the flagellar motor in *Bacillus subtilis*. The *B. subtilis* basal body consists of the C ring, MS ring and the rod and acts as a rotary motor. *Bacillus subtilis* flagellar motor has two distinct, MotAB and MotPS stators, which act as the H^+^ and Na^+^ channels, respectively. A dozen stators surround the rotor ring complex made of the MS ring formed by a transmembrane protein FliF and the C ring consisting of FliG, FliM and FliY^44^.
