# Supplementary material for: Load- and polysaccharide-dependent activation of the Na+-type MotPS stator in the Bacillus subtilis flagellar motor
Source: Sci Rep. 2017 Apr 5;7:46081. doi: 10.1038/srep46081 (PMC5380961; doi:10.1038/srep46081)
Supplement: Supplementary Information [file srep46081-s1.pdf]

Supplementary information

Load- and polysaccharide-dependent activation of the Na^+-type MotPS stator in the *Bacillus subtilis* flagellar motor

Naoya Terahara, Yukina Noguchi, Shuichi Nakamura, Nobunori Kami-ike, Masahiro Ito, Keiichi Namba and Tohru Minamino

Table S1. Rotational speed and torque of the MotAB motor.

	Bead size (μm)	2.0	1.5	1.0	0.8	0.6	0.5
NaCl	Speed (Hz)	10 ± 2	13 ± 2	47 ± 6	107 ± 15	155 ± 20	175 ± 33
	Torque (pN nm)	2,131 ± 278	2,081 ± 174	2,021 ± 205	1,879 ± 194	881 ± 182	535 ± 99
KCl	Speed (Hz)	8 ± 3	14 ± 3	56 ± 7	104 ± 12	166 ± 32	180 ± 36
	Torque (pN nm)	2,172 ± 337	2,244 ± 293	2,020 ± 315	1,801 ± 249	781 ± 112	485 ± 94

Table S2. Rotational speed and torque of the MotPS motor.

	Bead size (μm)	1.5	1.0	0.8	0.6	0.5
P_{motAB}	Speed (Hz)	1.4 ± 0.1	6 ± 1	11 ± 2	25 ± 5	33 ± 8
	Torque (pN nm)	220 ± 38	206 ± 35	191 ± 27	187 ± 39	118 ± 23
P_{grac}	Speed (Hz)	5 ± 1	19 ± 4	31 ± 4	37 ± 3	40 ± 4
	Torque (pN nm)	900 ± 236	790 ± 127	502 ± 54	313 ± 25	190 ± 16

Table S3. Rotational speed and torque of the MotPS motor measured using a 1.0-μm bead in media containing 0%, 2%, 4%, 6%, 8% and 10% Ficoll 400 (w/v).

Bead size (μm)	1.0	1.0	1.0	1.0	1.0	1.0
Ficoll (%)	0	2	4	6	8	10
Speed (Hz)	6 ± 1	7 ± 2	7 ± 2	6 ± 1	6 ± 1	5 ± 1
Torque (pN nm)	209 ± 35	331 ± 62	472 ± 124	645 ± 156	838 ± 185	1,150 ± 238

Table S4. Rotational speed and torque of the MotPS motor in media containing 10% Ficoll 400 (w/v).

Bead size (μm)	1.5	1.0	0.8	0.6	0.5
Ficoll (%)	10	10	10	10	10
Speed (Hz)	4 ± 2	6 ± 1	11 ± 3	24 ± 4	31 ± 6
Torque (pN nm)	1,717 ± 279	1,261 ± 238	995 ± 248	704 ± 118	563 ± 102

Table S5. Rotational speed and torque of the PS-L, PS-p2, and PS-p3 motors.

	Bead size (μm)	1.5	1.0	0.8	0.6	0.5
PS-L	Speed (Hz)	1.0 ± 0.6	6 ± 1	12 ± 2	26 ± 5	34 ± 7
	Torque (pN nm)	209 ± 71	217 ± 99	215 ± 70	203 ± 40	130 ± 20
PS-p2	Speed (Hz)	5 ± 1	19 ± 5	28 ± 4	35 ± 6	42 ± 6
	Torque (pN nm)	793 ± 221	652 ± 191	478 ± 64	289 ± 50	159 ± 19
PS-p3	Speed (Hz)	7 ± 2	25 ± 2	35 ± 6	41 ± 6	45 ± 6
	Torque (pN nm)	1,106 ± 127	860 ± 212	603 ± 87	359 ± 48	189 ± 22

Table S6. Rotational speed and torque of the AB-p3 motor.

	Bead size (μm)	2.0	1.5	1.0	0.8	0.6	0.5
NaCl	Speed (Hz)	10 ± 1	20 ± 2	40 ± 8	92 ± 19	149 ± 27	169 ± 31
	Torque (pN nm)	2,097 ± 396	2,271 ± 302	1,981 ± 281	1,895 ± 228	841 ± 142	585 ± 104
KCl	Speed (Hz)	8 ± 1	16 ± 4	32 ± 8	71 ± 14	123 ± 22	151 ± 36
	Torque (pN nm)	1,586 ± 373	1,599 ± 306	1,442 ± 285	1,414 ± 226	725 ± 134	500 ± 119

Table S7. Bacterial strains used in this study.

Strain	Relevant characteristics	Source or reference
Escherichia coli		
DH5αMCR	F^- *mcrA*Δ*1* (*mrr-hsd RMS-mcrBC*) Φ80*dlacZ* Δ(*lacZYAargF*) *U169 deoR recA1 endA1 supE44 λthi-1 gyr-496 relA1*	Stratagene
Salmonella		
SJW46	*fliC*(Δ205-293)	1
Bacillus subtilis		
BR151MA	*lys3 trpC2* (wild type)	2
Δhag	*lys3 trpC2* Δ*hag*::*spec*	This study
WT-sticky	*lys3 trpC2* Δ*hag*::*spec amyE*::P_{hag}-*hagsticky*	This study
ΔAB	*lys3 trpC2* Δ*motAB*::*ery*	3
ΔPS	*lys3 trpC2* Δ*motPS*::*neo*	3
ΔABΔPS	*lys3 trpC2* Δ*motAB*::*ery* Δ*motPS*::*neo*	3
AB	Δ*motAB* Δ*motPS amyE*::P_{motAB}-*motAB*	3
PS	Δ*motAB* Δ*motPS amyE*::P_{motAB}-*motPS*	3
AB-His_6	Δ*motAB* Δ*motPS amyE*::P_{motAB}-*motAB*-his_6	This study
PS-His_6	Δ*motAB* Δ*motPS amyE*::P_{motAB}-*motPS*-his_6	This study
P_{grac}-AB	Δ*motAB* Δ*motPS amyE*::P_{grac}-*motAB*	This study
P_{grac}-PS	Δ*motAB* Δ*motPS amyE*::P_{grac}-*motPS*	This study
ΔABΔPSΔHag	Δ*motAB* Δ*motPS* Δ*hag*::*spec*	This study
AB-sticky	Δ*motAB* Δ*motPS* Δ*hag*::*spec amyE*::P_{motAB}-*motAB*, P_{hag}-*hagsticky*	This study
PS-sticky	Δ*motAB* Δ*motPS* Δ*hag*::*spec amyE*::P_{motAB}-*motPS*, P_{hag}-*hagsticky*	This study
P_{grac}-AB-sticky	Δ*motAB* Δ*motPS* Δ*hag*::*spec amyE*::P_{grac}-*motAB*, P_{hag}-*hagsticky*	This study
P_{grac}-PS-sticky	Δ*motAB* Δ*motPS* Δ*hag*::*spec amyE*::P_{grac}-*motPS*, P_{hag}-*hagsticky*	This study
AB-p3-sticky	Δ*motAB* Δ*motPS* Δ*hag*::*spec amyE*::P_{motAB}-*motAB-p3*, P_{hag}-*hagsticky*	This study
PS-L-sticky	Δ*motAB* Δ*motPS* Δ*hag*::*spec amyE*::P_{motAB}-*motPS-L*, P_{hag}-*hagsticky*	This study
PS-p1-sticky	Δ*motAB* Δ*motPS* Δ*hag*::*spec amyE*::P_{motAB}-*motPS-p1*, P_{hag}-*hagsticky*	This study
PS-p2-sticky	Δ*motAB* Δ*motPS* Δ*hag*::*spec amyE*::P_{motAB}-*motPS-p2*, P_{hag}-*hagsticky*	This study
PS-p3-sticky	Δ*motAB* Δ*motPS* Δ*hag*::*spec amyE*::P_{motAB}-*motPS-p3*, P_{hag}-*hagsticky*	This study

References

1. Yoshioka, K., Aizawa, S.-I. & Yamaguchi, S. Flagellar filament structure and cell motility of *Salmonella typhimurium* mutants lacking part of the outer domain of flagellin. *J. Bacteriol.* **177**, 1090-1093 (1995).
2. Grundy, F.J., Turinsky, A.J. & Henkin, T.M. Catabolite regulation of *Bacillus subtilis* acetate and acetoin utilization genes by *CcpA*. *J. Bacteriol.* **176**, 4527-4533 (1994).
3. Ito, M., Terahara, N., Fujinami, S. & Krulwich, T.A. Properties of motility in *Bacillus subtilis* powered by the H^+-coupled MotAB flagellar stator, Na^+-coupled MotPS or hybrid stators MotAS or MotPB. *J. Mol. Biol.* **352**, 396–408 (2005).

Table S8. Plasmids used in this study.

Plasmid	Relevant characteristics	Source or reference
pDG1730	*amyE* integration vector with *Spec*^{r} gene	1
pDR67	*amyE* integration vector with *Cm*^{r} gene and P_{spac} promoter upstream of multiple cloning site	2
pHT01	*B. subtilis* expression vector by P_{grac} promoter	MoBiTec
pDR-AB	pDR67 + P_{motAB}-*motAB* from BR151MA	3
pDR-PS	pDR67 + P_{motAB}-*motPS* from BR151MA	3
pDR-AB-His$_6$	pDR67 + P_{motAB}-*motAB-his*$_6$	This study
pDR-PS-His$_6$	pDR67 + P_{motAB}-*motPS-his*$_6$	This study
pHT-AB	pHT01 + P_{grac}-*motAB*	This study
pHT-PS	pHT01 + P_{grac}-*motPS*	This study
pDR-P_{grac}-AB	pDR67 + P_{grac}-*motAB*	This study
pDR-P_{grac}-PS	pDR67 + P_{grac}-*motPS*	This study
pDR-hagsticky	pDR67 + P_{hag}-*hagsticky* from BR151MA and SJW46	This study
pDR-ABsticky	pDR-hagsticky + P_{motAB}-*motAB*	This study
pDR-PSsticky	pDR-hagsticky + P_{motAB}-*motPS*	This study
pDR- P_{grac}-ABsticky	pDR-hagsticky + P_{grac}-*motAB*	This study
pDR- P_{grac}-PSsticky	pDR-hagsticky + P_{grac}-*motPS*	This study
pDR-ABp3sticky	pDR-hagsticky + P_{motAB}-*motAB-p3*	This study
pDR-PSLsticky	pDR-hagsticky + P_{motAB}-*motPS-L*	This study
pDR-PSp1sticky	pDR-hagsticky + P_{motAB}-*motPS-p1*	This study
pDR-PSp2sticky	pDR-hagsticky + P_{motAB}-*motPS-p2*	This study
pDR-PSp3sticky	pDR-hagsticky + P_{motAB}-*motPS-p3*	This study

References

1. Guérout-Fleury, A.M., Frandsen, N. & Stragier, P. Plasmids for ectopic integration in *Bacillus subtilis*. *Gene* **180**, 57-61 (1996).
2. Ireton, K., Rudner, D.Z., Siranosian, K.J. & Grossman, A.D. Integration of multiple developmental signals in *Bacillus subtilis* through the Spo0A transcription factor. *Genes & Dev.* **7**, 283-294 (1993).
3. Ito, M., Terahara, N., Fujinami, S. & Krulwich, T.A. Properties of motility in *Bacillus subtilis* powered by the H^+-coupled MotAB flagellar stator, Na^+-coupled MotPS or hybrid stators MotAS or MotPB. *J. Mol. Biol.* **352**, 396–408 (2005).

Table S9. Oligonucleotides used in this study.

Primer	Sequence (5’ → 3’)
Hag-del-F1	ATACTCCGTCACAGCTTGATGTGC
Hag-del-F2	CCTCACAAAAAAAGTGAGGATTATGAGCAATTTGATTAACGGAA
Hag-del-F3	ATTCTTTGCCAGAACTAATTGATTTTAAAAAAGACCTTGGCGTT
Hag-del-R1	CGCCTTGTGTGTATCCCGTATAAT
Hag-del-R2	TTCCGTTAATCAAATTGCTCATAATCCTCACTTTTTTTGTGAGG
Hag-del-R3	AACGCCAAGGTCTTTTTTAAAATCAATTAGTTCTGGCAAAGAAT
Hag-*Bam*HI-F	CGGGATCCCGAAGCGATTCAAATAGGTGCTGA
Hag-sticky-F1	GGTAAGAAATTGCTCGATGGCACTAACACCCTGACCATCCAGGTTGGT
Hag-sticky-F2	GGTCACAACTTTAAAGCACAGCCTGATGCTCAATTTGAAAGTTGTTGAT
Hag-*Sph*I-R	ACATGCATGCGGATGAGGAATGATTAGGAGATAG
Hag-sticky-R1	ACCAACCTGGATGGTCAGGGTGTTAGTGCCATCGAGCAATTTCTTACC
Hag-sticky-R2	ATCAACAACTTTCAATTGAGCATCAGGCTGTGCTTTAAAGTTGTGACC
AB-*Xma*I-F	TCCCCCCGGGCTTCTCCTGAAGCGCTACTTT
AB-*Bam*HI-F	CGGGATCCATGGATAAAACTTCGTTAATCGGT
AB-*SphI*-R	ACATGCATGCAAAACAGTTTCATAATAAGTAA
AB-His$_6$-*SphI*-R	ACATGCATGCCTAGTGGTGGTGGTGGTGGTGTTTTTCATTTGTTTCCGCTGCGC
AB-*Xma*I-R	TCCCCCCGGGAAGCCTGATATGTCACAAGGC
PS-*Bam*HI-F	CGGGATCCATGAAACGTTTTGATTATCTTACA
PS-*Sph*I-R	ACATGCATGCCCAAACTGTCAGCAAAGCAT
PS-His$_6$-*Sph*I-R	ACATGCATGCTTAGTGGTGGTGGTGGTGGTGCGAAGAGGTCGTTTTTGATTTTT
PS-*Xma*I-R	TCCCCCCGGGAGCGATCCGGCTGATTTATAT
P$_{grac}$-*Xma*I-F	TCCCCCCGGGTTTAAATGCAACCGTTTTTTC
AB-p3-F	TGAAGGCCTTCTGATTACGATTCAGGAGGCTGTGCTGTTTGATA
AB-p3-R	TATCAAACAGCACAGCCTCCTGAATCGTAATCAGAAGGCCTTCA
PS-L-F1	GAAAGATCTTGTGAAAACCTTTTCTGAATGGGCTCAGCTTGCAC
PS-L-F2	AAACGGGCTCAGCATGGCTGTTGACGGCTGGGATGAAGAAACGA
PS-L-R1	GTGCAAGCTGAGCCCATTCAGAAAAGGTTTTCACAAGATCTTTC
PS-L-R2	TCGTTTCTTCATCCCAGCCGTCAACAGCCATGCTGAGCCCGTTT
PS-p1-F	CTTTTCGATGTCCCAAATCGATGCAGCTAAGTTTCAAATGCTCT
PS-p1-R	AGAGCATTTGAAACTTAGCTGCATCGATTTGGGACATCGAAAAG
PS-p2-F	ATCTGATACGAAGAAGCAAGAGGAACTTGAAAATGTGAAGAGCC
PS-p2-R	GGCTCTTCACATTTTCAAGTTCCTCTTGCTTCTTCGTATCAGAT
PS-p3-F	ACGCGGTGTCGTGCTCGTTCTTAAAGACAGCATCTTCTTCGATT
PS-p3-R	AATCGAAGAAGATGCTGTCTTTAAGAACGAGCACGACACCGCGT
